# Supplementary material for: Selective Removal of Chlorophyll and Isolation of Lutein from Plant Extracts Using Magnetic Solid Phase Extraction with Iron Oxide Nanoparticles
Source: Int J Mol Sci. 2024 Mar 9;25(6):3152. doi: 10.3390/ijms25063152 (PMC10970386; doi:10.3390/ijms25063152)
Supplement: Supplementary file 1 [file ijms-25-03152-s001.zip › ijms-2891963-supplementary.pdf]

Supplemental information

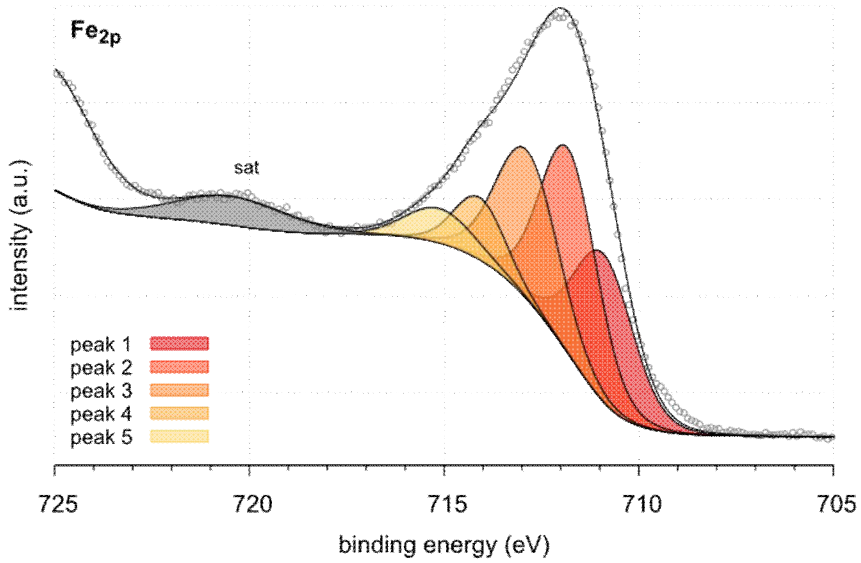

Figure S1. Fe_{2p} spectrum fitted using FeOOH model described by Biesinger [43].

Table S1. The results of fitting of Fe_{2p} spectrum with FeOOH peaks: binding energies (BE), peak widths (eV) and their intensities (%). Model based on publication by Biesinger [43].

**Sample**	**Peak**	**BE (eV)**	**FWHM (eV)**	**Intensity (%)**
Fe-nps	FeOOH peak 1	710.9	1.80	22.9
	FeOOH peak 2	711.8	1.73	30.5
	FeOOH peak 3	712.9	1.87	24.0
	FeOOH peak 4	714.1	1.54	8.2
	FeOOH peak 5	715.1	2.05	6.1
	sat 1	720.5	3.39	8.3

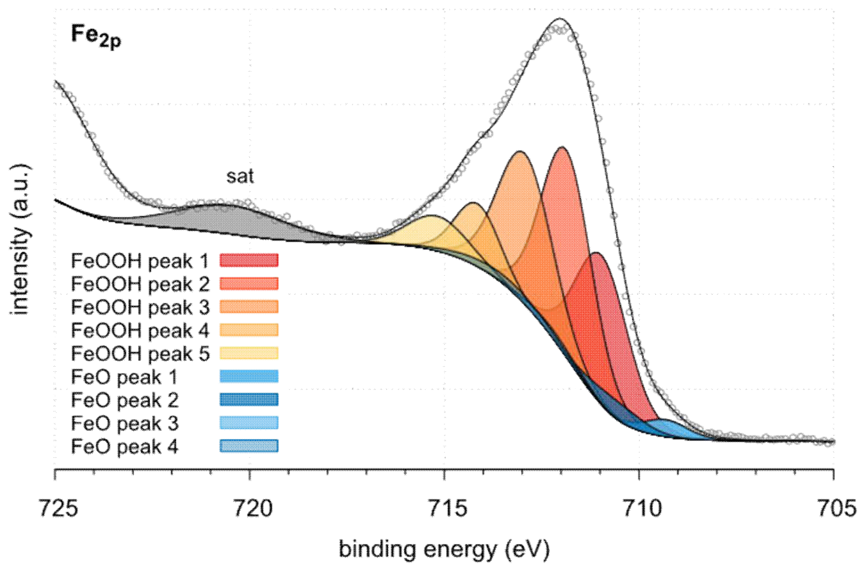

Figure S2. Fe_{2p} spectrum fitted using FeOOH and FeO model described by Biesinger [43].

Table S2. The results of fitting of Fe_{2p} spectrum with FeOOH and FeO peaks: binding energies (BE), peak widths (eV) and their intensities (%). Model based on publication by Biesinger [43].

Sample	Peak	BE (eV)	FWHM (eV)	Intensity (%)
Fe-nps	FeO peak 1	709.3	1.37	2.1
	FeO peak 2	710.6	1.63	2.7
	FeO peak 3	712.2	1.64	1.3
	FeO peak 4	713.4	2.91	2.3
	FeOOH peak 1	711.0	1.60	20.8
	FeOOH peak 2	711.9	1.54	27.8
	FeOOH peak 3	712.9	1.66	21.8
	FeOOH peak 4	714.2	1.37	7.5
	FeOOH peak 5	715.2	1.82	5.6
	sat 1	720.5	3.39	8.2
